# H3K4me3 and H3K27ac Promote ccRCC Proliferation Through the CDC6-EXOSC5 Axis

**DOI:** 10.3390/ijms27135657

**Published:** 2026-06-23

**Authors:** Peng Cui, Juan Luo, Ping Zhang, Qiongye Dong, Xiangling Chen

**Affiliations:** 1 Precision Medicine Institute, Peking University Shenzhen Hospital, Shenzhen 518036, China; cuipeng279@163.com (P.C.); luojuan1010@163.com (J.L.); 2 Intervention and Cell Therapy Center, Peking University Shenzhen Hospital, Shenzhen 518036, China; 3 Department of Hematology, Peking University Shenzhen Hospital, Shenzhen 518036, China; zhangping203@163.com; 4 Department of Urology, Peking University Shenzhen Hospital, Shenzhen 518036, China

**Keywords:** clear cell renal cell carcinoma, CDC6, SETD1A, EXOSC5, proliferation

## Abstract

Renal cell carcinoma (RCC) is one of the most common malignant tumors of the urinary system, with clear cell renal cell carcinoma (ccRCC) accounting for more than 75% of RCC cases and representing the primary cause of mortality in renal cancer patients. CDC6 exhibits oncogenic characteristics and plays a significant role in tumor grading and prognosis prediction. Analysis of The Cancer Genome Atlas (TCGA) data shows that the CDC6 gene is significantly overexpressed in 97.22% (70/72) of paired clinical samples in ccRCC tissues compared to adjacent normal tissues. Consistent with this, elevated CDC6 protein levels were observed across all four paired tumor tissues examined. Functional experiments further confirm that CDC6 expression levels directly influence cellular proliferation, as its knockdown suppresses cell viability by ~60% in CCK-8 assays (*p* < 0.001) and reduces EdU incorporation by ~50%. Mechanistically, in tumor tissues, CDC6 transcription is epigenetically regulated by histone acetylation and methylation, which in turn modulates downstream effectors, e.g., the exosome complex protein EXOSC5. Our findings indicate that in ccRCC, increased histone H3K4 trimethylation near the CDC6 transcriptional start site enhances its expression. The methyltransferase SETD1A may act as a potential upstream regulator mediating the transcriptional activation of CDC6, thereby driving tumor progression through the regulation of EXOSC5. We have further investigated the relationship between the CDC6-associated gene network and tumor development and clarified the diagnostic and prognostic relevance of the SETD1A–CDC6–EXOSC5 axis in ccRCC. The outcomes of this research are expected to provide novel insights into the pathogenesis of renal cell carcinoma and establish a theoretical foundation for new diagnostic strategies.

## 1. Introduction

Renal cell carcinoma (RCC), commonly referred to as kidney cancer, originates from renal tubular epithelial cells and accounts for approximately 80–90% of kidney tumors [1]. Clear cell renal cell carcinoma (ccRCC) accounts for 75% of RCC cases and is the primary cause of death in patients with kidney cancer [2,3]. Localized or locally advanced RCC is amenable to surgical resection, yet the early stages of kidney cancer are typically asymptomatic, and the disease remains prone to recurrence. Approximately 20–30% of patients are diagnosed with distant metastasis upon initial detection, rendering curative surgical resection unfeasible [4]. For these patients with metastatic ccRCC, the 5-year survival rate remains as low as 10% [5,6]. Once metastasis occurs, the disease shows poor response to conventional radiotherapy and chemotherapy, and resistance to targeted therapeutic agents frequently emerges, which constitutes the primary cause of mortality in kidney cancer patients [7]. Given the complex pathogenesis of ccRCC and its inherent resistance to standard systemic therapies, the limited availability of actionable therapeutic targets continues to be a major bottleneck in clinical management. Therefore, investigating the underlying molecular mechanisms of ccRCC progression is essential for identifying reliable biomarkers for early diagnosis and novel targets for therapeutic intervention.

Epigenetics plays a significant role in the tumorigenesis and immune regulation of kidney cancer [8,9]. Histone modifications, as a key form of epigenetic regulation, exert important influences on gene expression [10]. Among them, methylation and acetylation are the most common types of histone modifications [11]. Enzymes regulating histone modifications include histone acetyltransferases (HATs), histone deacetylases (HDACs), histone methyltransferases (HMTs), and histone demethylases (HDMTs). These enzymes play crucial roles in modulating chromatin states, thereby controlling transcriptional processes and maintaining the chromatin configuration necessary for cell proliferation, which facilitates transcription [12]. Studies have reported that enzymes involved in acetylation and methylation modifications participate in multiple physiological processes that contribute to renal diseases, such as renal fibrosis, acute kidney injury, and kidney tumorigenesis [13,14,15]. For example, our group previously demonstrated that NUF2 is epigenetically upregulated by CBP and SMYD3, promoting ccRCC progression via the KDM2A-HMGA2 axis [16,17]. Thus, research on histone-modifying enzymes holds promise for providing novel targets for the diagnosis and treatment of kidney diseases. SET1A, also known as SETD1A or KMT2F, is a member of the KMT2 family. Its biological functions are typically attributed to its methyltransferase activity on histone H3 lysine 4 (H3K4). H3K4 methylation can exist in mono-, di-, or trimethylated states (Me1, Me2, Me3), with H3K4Me3 highly enriched at gene promoters to facilitate transcription [18]. SETD1A activity has been shown to be inhibited by ABHD5 in colorectal cancer [19]. GAU1 forms a triplex structure to recruit SETD1A, triggering H3K4me3-mediated SENP5 transcriptional activation and tumorigenesis [20]. While SETD1A’s role in cancer is established, its contribution to ccRCC pathogenesis constitutes a gap in knowledge. Preliminary findings from our laboratory demonstrate that CDC6 is highly expressed and harbors H3K4me3 marks in RCC, prompting us to investigate whether SETD1A mediates this specific epigenetic activation.

Cell division control protein 6 (CDC6) plays a critical role in the early stage of DNA replication and cell cycle regulation. CDC6 interacts with DNA replication licensing factors MCM, the origin recognition complex (ORC), chromatin, and the licensing factor CDT1. This classical complex, ORC–CDC6–CDT1–MCM2-7, assembles during the initiation of DNA replication and regulates DNA replication, thereby influencing the cell cycle and cell division [21,22]. Given its key function in cell cycle control, CDC6 exhibits oncogenic characteristics and is significantly overexpressed in various solid tumors. For instance, in hepatocellular carcinoma, prostate cancer and bladder cancer, CDC6 expression is regulated by histone modifications [23,24] and deubiquitinase OTUD6A [25]. Through bioinformatics analysis of TCGA data, researchers have revealed that CDC6 mRNA expression is elevated in renal cancer tissues [26], and its high expression promotes cell proliferation and metastasis [27]. Thus, while CDC6 is established as a crucial driver of tumorigenesis, the precise epigenetic mechanisms governing its overexpression and pro-tumorigenic functions in ccRCC remain to be fully elucidated.

Using approaches such as affinity mass spectrometry and quantitative proteomics, we identified EXOSC5 as a potential downstream target of CDC6. EXOSC5 (Exosome complex component RRP46) is a component of the exosome complex, a conserved ribonuclease complex composed of ten evolutionarily conserved subunits. It processes and degrades various RNA molecules in both the nucleus and cytoplasm. EXOSC5 has been reported to maintain tumor stemness in endometrial cancer [28], and to regulate the development of colorectal cancer via the ERK-AKT signaling pathway [29] as well as gastric cancer through the AKT/STAT3 pathway [30]. However, little is known about the function of EXOSC5 in ccRCC.

Collectively, we sought to test the central hypothesis that the SETD1A–CDC6–EXOSC5 axis represents a previously unrecognized epigenetic driver in ccRCC, offering new insights into its pathogenesis and prognostic stratification.

## 2. Results

### 2.1. High CDC6 Expression Correlates with Aggressive Clinicopathological Features and Poor Survival in ccRCC

**Figure 1:**
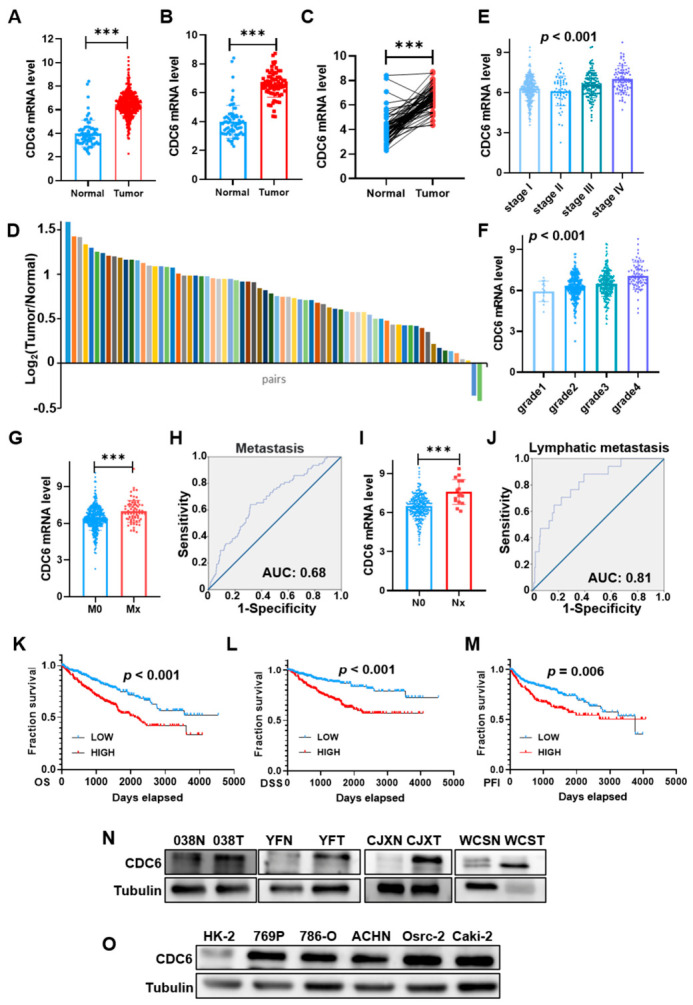
CDC6 is upregulated and serves as a prognostic biomarker in clear cell renal cell carcinoma. (**A**–**D**) CDC6 mRNA expression levels in ccRCC tumor tissues versus adjacent normal tissues, analyzed using data from TCGA (**A**); highly expressed in 72 paired samples (**B**); scatter diagram of 72 paired samples (**C**); Log_2_-transformed fold change of CDC6 expression (tumor/normal) in paired ccRCC patient samples. Each bar of a distinct color represents one case. A total of 84 paired samples from 84 cases are included. (**D**); (**E**,**F**) CDC6 expression across different tumor stages (**E**) and pathological grades (**F**) in ccRCC patients; (**G**,**I**) CDC6 expression levels in patients with (Mx/Nx) and without (M0/N0) distant metastasis and lymphatic metastasis. (**H**,**J**) Receiver operating characteristic (ROC) curves showing the diagnostic value of CDC6 for predicting distant metastasis (AUC = 0.68) and lymphatic metastasis (AUC = 0.81). AUC values stands for Area Under the Curve. (**K**–**M**) Kaplan–Meier survival analysis of overall survival (OS, (**K**)), disease-specific survival (DSS, (**L**)), and progression-free interval (PFI, (**M**)) in ccRCC patients stratified by CDC6 expression level (high vs. low). (**N**,**O**) Western blot analysis of CDC6 protein expression in paired clinical ccRCC tissues (**N**) and different ccRCC cell lines (**O**). All data are presented as mean ± SD. *** *p* < 0.001.

To clarify the clinical significance of CDC6 in clear cell renal cell carcinoma (ccRCC), we analyzed its expression using TCGA-ccRCC data. As shown in Figure 1A, CDC6 mRNA levels were significantly higher in tumor tissues than in adjacent normal tissues. This trend was consistently observed in 72 paired samples, visualized via a strip plot (Figure 1B) and a paired scatter diagram (Figure 1C). While most paired samples exhibited concordant overexpression, a small proportion showed inconsistent expression trends between tumor and normal tissues. A bar chart of log_2_(Tumor/Normal) fold-change further illustrated that CDC6 expression was decreased in only 2 of 72 paired samples (≈2.78%), corresponding to a positive rate of CDC6 overexpression in tumors of 97.22% (Figure 1D). CDC6 expression was significantly elevated in patients with advanced primary tumor stage (Figure 1E), higher histological grade (Figure 1F), distant metastasis (Figure 1G), and lymph node metastasis (Figure 1I). ROC analysis demonstrated that CDC6 could discriminate metastatic status with AUC values of 0.68 for distant metastasis (Figure 1H) and 0.81 for lymph node metastasis (Figure 1J), indicating high accuracy, especially for identifying lymph node-positive cases. We further examined the prognostic value of CDC6 in ccRCC. Based on the gene expression levels detected in the cohort, patients were dichotomized into high- and low-expression groups using the median as the cutoff. Log-rank survival analysis revealed that high CDC6 expression was associated with shorter overall survival (Figure 1K), disease-specific survival (Figure 1L), and progression-free survival (Figure 1M), supporting its prognostic value. Western blot analysis of four paired clinical samples confirmed upregulation of CDC6 protein in ccRCC tissues compared with adjacent normal tissues (Figure 1N). Consistently, elevated CDC6 protein levels were also detected in ccRCC cell lines relative to the normal renal tubular epithelial cell line HK-2 (Figure 1O). Collectively, these results demonstrate that high CDC6 expression correlates with aggressive clinicopathological features and poorer survival outcomes, suggesting its potential as a diagnostic biomarker and prognostic indicator for risk stratification in ccRCC patients.

### 2.2. CDC6 Promotes the Proliferation of Clear Cell Renal Cell Carcinoma Cells

**Figure 2:**
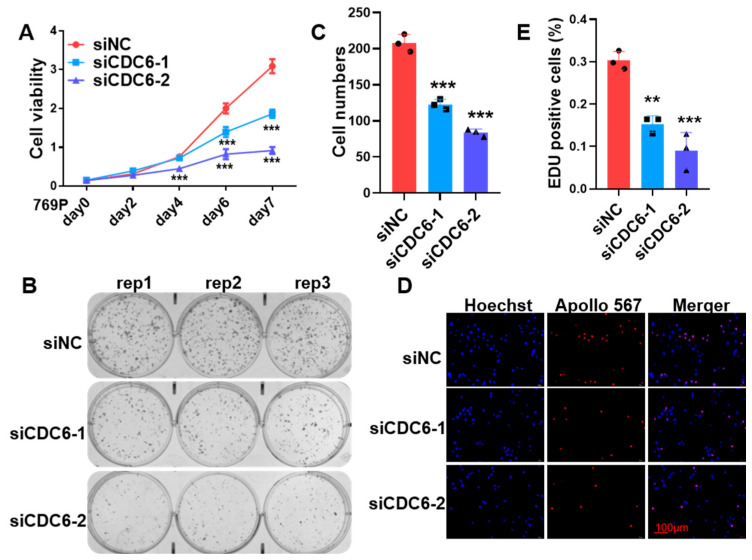
Knockdown of CDC6 inhibits the proliferation of 769P clear cell renal cell carcinoma cells. (**A**) Cell viability of 769P cells transfected with siNC, siCDC6-1, or siCDC6-2 was assessed by CCK-8 assay over 7 days. (**B**) Representative images of colony formation assays in 769P cells after CDC6 knockdown (three biological replicates). (**C**) Quantification of colony numbers in the colony formation assay by bar graph with overlaid individual data points. (**D**) Representative images of EdU incorporation assay (scale bar: 100 μm; Hoechst: blue, nuclei; Apollo 567: red, proliferating cells). (**E**) Quantification of EdU-positive cell ratio in each group by bar graph with overlaid individual data points. Data are presented as mean ± SD; ** *p* < 0.01, *** *p* < 0.001.

**Figure 3:**
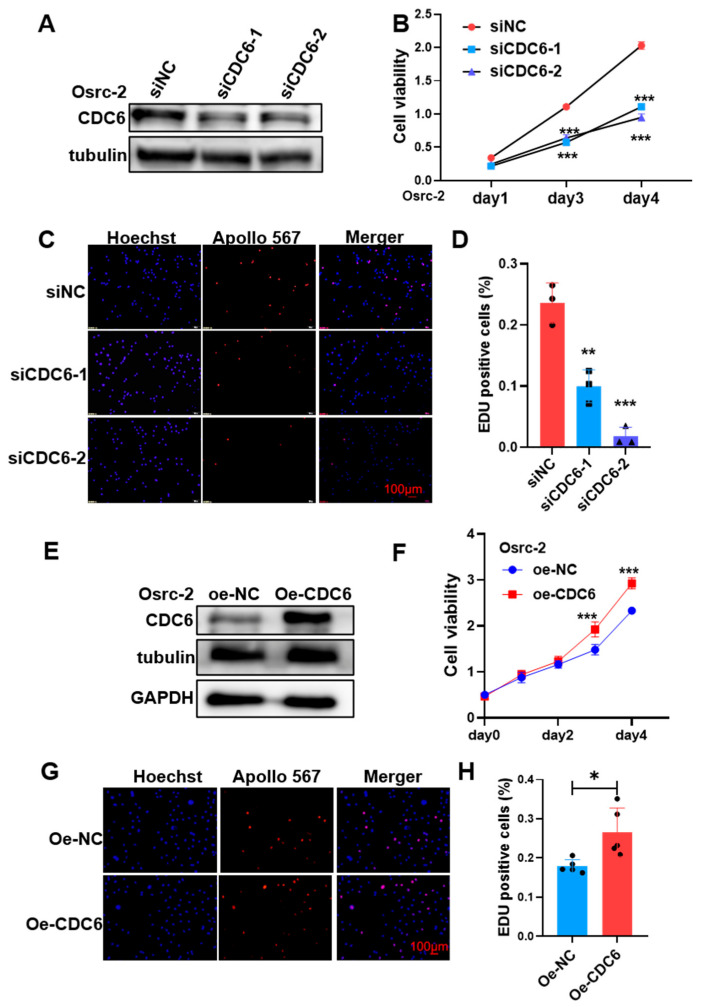
CDC6 modulates the proliferation of Osrc-2 clear cell renal cell carcinoma cells. (**A**) Western blot validation of CDC6 knockdown efficiency in Osrc-2 cells transfected with siNC, siCDC6-1, or siCDC6-2. (**B**) CCK-8 cell viability assay showing reduced proliferation in CDC6-knockdown Osrc-2 cells. (**C**,**D**) Representative images and quantification of EdU incorporation assay by bar graph with overlaid individual data points, showing decreased EdU-positive cells in CDC6-silenced Osrc-2 cells. (**E**) Western blot validation of CDC6 overexpression efficiency in Osrc-2 cells. (**F**) CCK-8 cell viability assay showing enhanced proliferation in CDC6-overexpressing Osrc-2 cells. (**G**,**H**) Representative images and quantification of EdU incorporation assay by bar graph with overlaid individual data points, showing increased EdU-positive cells in CDC6-overexpressing Osrc-2 cells. Data are presented as mean ± SD; * *p* < 0.05, ** *p* < 0.01, *** *p* < 0.001.

To investigate the role of CDC6 in ccRCC, we first conducted an analysis using the STRING database, which revealed that CDC6 interacts with DNA replication licensing factors, including the mini chromosome maintenance complex (MCM), the origin recognition complex (ORC), and chromatin licensing and DNA replication factor 1 (CDT1) (Supplementary Figure S1A). These interactions contribute to the formation of the pre-replication complex ORC–CDC6–CDT1–MCM2-7, which is involved in regulating DNA replication, cell cycle progression, and cell division [21,22,31,32]. CDK2, a known kinase reported to phosphorylate CDC6 [33]. In our study, western blot analysis in 769P and Osrc-2 cells showed that knockdown of CDC6 led to a concomitant decrease in CDK2 protein levels (Supplementary Figure S1B,C). Given the critical role of CDC6-CDK2 in cell cycle regulation, DNA replication, and DNA damage response [34,35], we next examined the effect of CDC6 on cell proliferation. In 769P cells, knockdown of CDC6 significantly attenuated proliferative capacity, as demonstrated by CCK-8 assay (Figure 2A), colony formation assay (Figure 2B,C), and EdU incorporation assay (Figure 2D,E). Similarly, in Osrc-2 cells, siRNA-mediated knockdown or overexpression of CDC6 was confirmed by Western blot (Figure 3A,E). Functional assays showed that CDC6 knockdown markedly inhibited cell proliferation (Figure 3B–D), whereas CDC6 overexpression enhanced proliferative ability (Figure 3F–H). Consistent with these findings, CDC6 knockdown also suppressed proliferation in 786-O and ACHN cells (Supplementary Figure S2A,B). Taken together, these results indicate that CDC6 plays an important role in regulating the proliferation of ccRCC cells.

### 2.3. CDC6 Knockdown Downregulates EXOSC5

**Figure 4:**
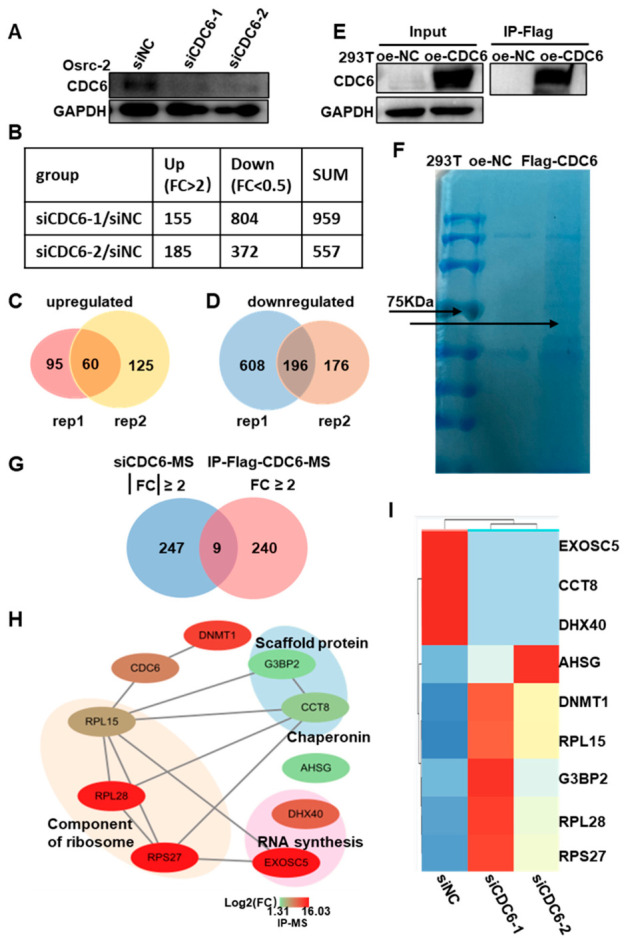
Identification of CDC6-associated proteins via label-free MS proteomic and interactomic analyses. (**A**) Western blot validation of CDC6 knockdown efficiency in Osrc-2 cells. (**B**) Summary table of differentially expressed proteins (DEPs) in CDC6-knockdown cells. The numbers of significantly upregulated (FC > 2) and downregulated (FC < 0.5) proteins in siCDC6-1/siNC and siCDC6-2/siNC comparisons are listed. (**C**,**D**) Venn diagrams showing the overlap of DEPs between two biological replicates. Upregulated proteins (**C**) and downregulated proteins (**D**) from two independent knockdown experiments (rep1 and rep2) were analyzed to identify consistent changes. (**E**) Validation of CDC6 overexpression and immunoprecipitation (IP) efficiency in 293T cells. (**F**) SDS-PAGE and Coomassie blue staining of immunoprecipitated Flag-CDC6 protein. Purified Flag-CDC6 protein from 293T cells was separated by SDS-PAGE. (**G**) Venn diagram showing the overlap between CDC6-regulated proteins and CDC6-interacting proteins. Nine proteins were found in both datasets. (**H**) Functional interaction network of CDC6 and its candidate binding partners. (**I**) Heatmap showing expression changes of key candidate proteins upon CDC6 knockdown. The color gradient indicates the direction of change, with red representing upregulation and blue representing downregulation.

**Figure 5:**
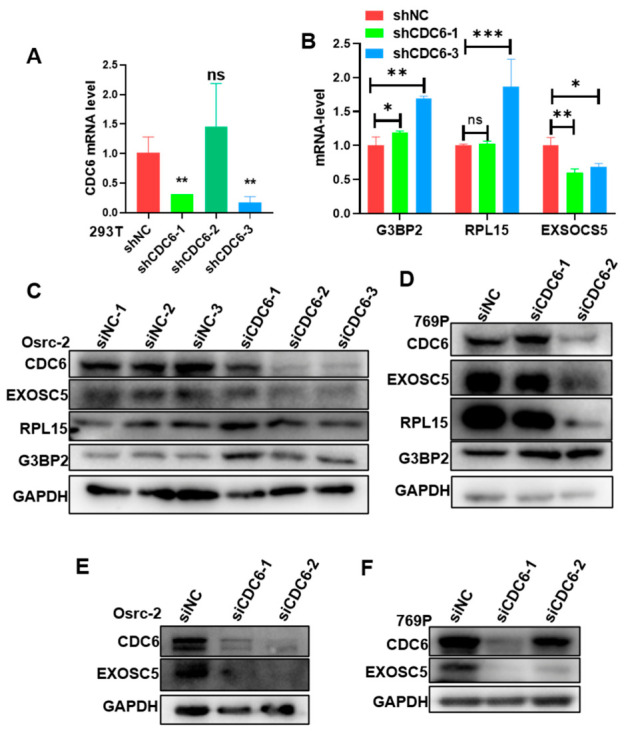
Validation of CDC6 knockdown and its effect on the identified proteins. (**A**) CDC6 mRNA levels in 293T cells after shRNA-mediated knockdown. Cells were transduced with non-targeting shRNA (shNC) or two independent CDC6-targeting shRNAs (shCDC6-1, shCDC6-3). CDC6 mRNA expression was measured by qRT-PCR. shCDC6-2 served as a non-effective control. (**B**) mRNA expression of CDC6-regulated proteins upon CDC6 knockdown in 293T cells. qRT-PCR analysis of G3BP2, RPL15, and EXOSC5 mRNA levels in shNC, shCDC6-1, and shCDC6-3 cells. (**C**,**D**) Western blot analysis of CDC6 and its regulated proteins in Osrc-2 (**C**) and 769P (**D**) cells after siRNA-mediated CDC6 knockdown. (**E**,**F**) Western blot validation of CDC6 knockdown efficiency and its effect on EXOSC5 expression in Osrc-2 (**E**) and 769P (**F**) cells. Data are presented as mean ± SD (*n* = 3). * *p* < 0.05; ** *p* < 0.01; *** *p* < 0.001; ns, not significant.

To investigate the mechanism of action of CDC6, we performed label-free quantitative proteomic analysis on Osrc-2 cells transfected with two independent siRNAs targeting CDC6, alongside a negative control (siNC) group (Figure 4A). In the first group, 155 proteins were upregulated and 804 were downregulated upon CDC6 knockdown; in the second group, 185 proteins were upregulated and 372 were downregulated (Figure 4B and Supplementary Table S1). Combined analysis of the two groups identified 60 consistently upregulated proteins (Figure 4C) and 196 consistently downregulated proteins (Figure 4D). Meanwhile, we overexpressed CDC6 in 293T cells and performed co-immunoprecipitation, separately immunoprecipitating the CDC6 overexpression and empty vector control groups (Figure 4E) and followed by SDS-PAGE, Coomassie staining, in-gel digestion, and mass spectrometry (Figure 4F). This identified 249 potential CDC6-interacting proteins (Figure 4G and Supplementary Table S2). Intersecting the proteins from the label-free proteomics and affinity purification-mass spectrometry datasets yielded nine proteins that are both regulated by and interact with CDC6. The color gradient in the network reflects their binding strength to CDC6 (Figure 4H). Among them, ribosomal protein RPL15 emerged as a potential hub protein. Notably, EXOSC5, a component of the exosome complex involved in RNA metabolism, showed the strongest binding, and G3BP2, a GTPase-activating protein-binding protein participating in multiple biological processes, also attracted our attention. The expression intensities of these nine proteins in CDC6-knockdown versus control Osrc-2 cells are shown in Figure 4I. Mass spectrometry data revealed that upon CDC6 depletion, RPL15 and G3BP2 were upregulated, whereas EXOSC5 was downregulated (Figure 4I). We then used RT-PCR to examine the mRNA levels of RPL15, G3BP2, and EXOSC5 in 293T cells. CDC6 knockdown was validated in Figure 5A, with shCDC6-1 and shCDC6-3 showing efficient knockdown. The mRNA levels of G3BP2 and EXOSC5 mirrored the protein-level changes, while RPL15 exhibited inconsistent trends under two different shRNAs (Figure 5B). We further performed Western blot analysis to assess the protein levels of RPL15, G3BP2, and EXOSC5 in clear cell renal cell carcinoma lines 769P and Osrc-2. Using previously collected knockdown samples, we found that in Osrc-2 cells, EXOSC5 was downregulated upon CDC6 knockdown, G3BP2 and RPL15 displayed a slight upward trend (Figure 5C). In 769P cells, CDC6 knockdown led to downregulation of EXOSC5 and a decreasing trend for RPL15, while G3BP2 displayed a slight upward trend (Figure 5D). To further confirm the alteration of EXOSC5, we repeated the infection in Osrc-2 and 769P cells. Consistently, CDC6 knockdown resulted in downregulation of EXOSC5 in both cell lines (Figure 5E,F). Therefore, EXOSC5 represents another downstream regulatory protein of CDC6.

### 2.4. EXOSC5 Is Upregulated in ccRCC and Correlated with Various Clinicopathological Variables

**Figure 6:**
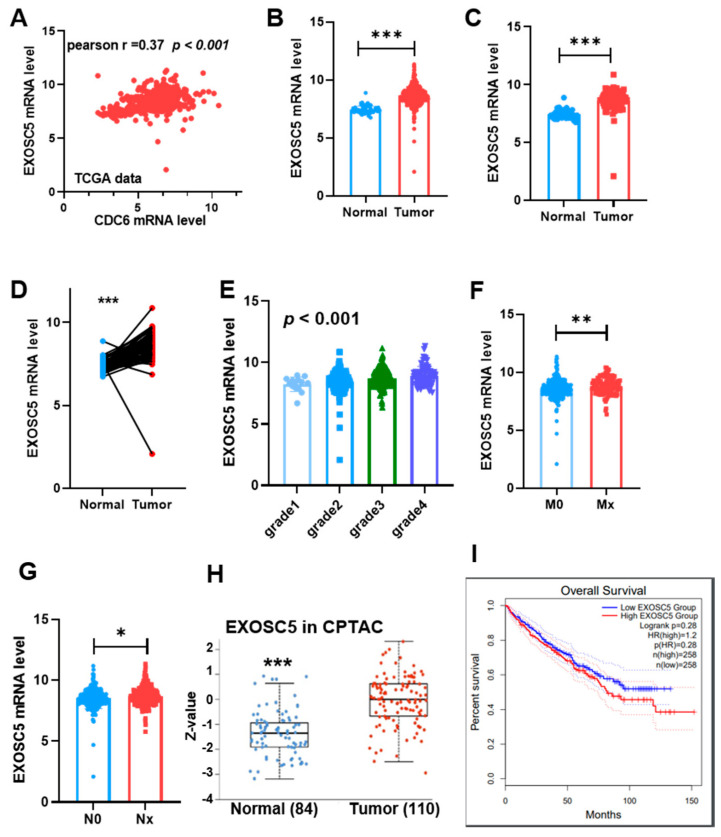
EXOSC5 is upregulated in ccRCC. (**A**) Pearson correlation analysis showed a significant positive correlation between CDC6 and EXOSC5 mRNA levels (r = 0.37, *p* < 0.001). (**B**) EXOSC5 expression was significantly upregulated in tumor tissues compared with adjacent normal tissues. (**C**,**D**) Paired analysis of EXOSC5 expression in matched normal and tumor samples. (**E**) EXOSC5 expression was significantly correlated with pathological grade. (**F**,**G**) EXOSC5 mRNA levels were significantly higher in patients with advanced metastasis (**F**) and lymph metastasis (**G**). (**H**) EXOSC5 protein expression in normal and tumor tissues from the CPTAC dataset. (**I**) Kaplan–Meier survival analysis of overall survival based on EXOSC5 expression. Data are presented as mean ± SD (*n* = 3). * *p* < 0.05; ** *p* < 0.01; *** *p* < 0.001.

Based on the above mechanism, given that CDC6 regulates EXOSC5, we further investigated their potential clinical relevance. First, we analyzed the expression correlation between EXOSC5 and CDC6, as well as the clinical characteristics of EXOSC5 in ccRCC. As shown in Figure 6A, EXOSC5 and CDC6 expression were positively correlated. EXOSC5 was upregulated at both the mRNA (Figure 6B) and protein levels (Figure 6H) in tumor tissues. This trend was consistent in 72 paired samples (Figure 6C), and a paired scatter plot clearly showed that 69 of the 72 pairs (96%) exhibited upregulation in tumor tissues (Figure 6D). EXOSC5 expression was significantly elevated in patients with higher histological grade (Figure 6E), distant metastasis (Figure 6F), and lymph node metastasis (Figure 6G). Patients with high expression of EXOSC5 alone tended to have poorer overall survival, though the difference was not statistically significant (Figure 6I). In summary, EXOSC5 is upregulated in ccRCC and closely associated with adverse pathological features, suggesting its potential value as an oncogene and a prognostic biomarker. 

### 2.5. Histone H3K27ac and H3K4me3 Enrichment Upregulates CDC6 Expression

**Figure 7:**
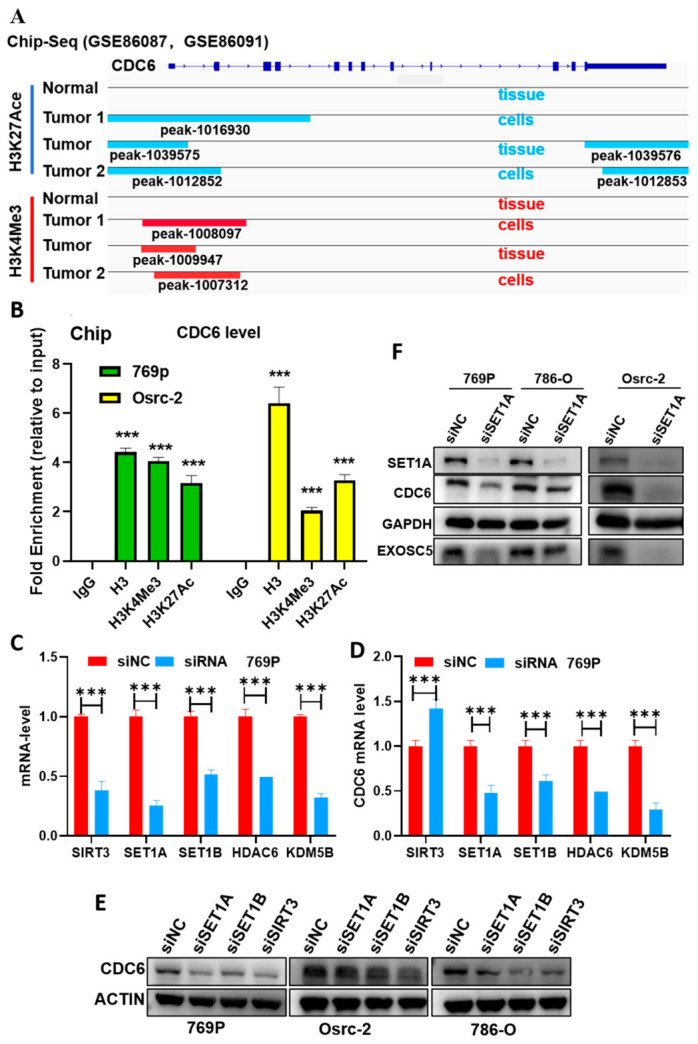
CDC6 transcription is regulated by SETD1A in renal cancer cells. (**A**) Enrichment profiles of H3K27Ac and H3K4Me3 at the CDC6 promoter locus based on public ChIP-Seq datasets (GSE86087, GSE86091). (**B**) ChIP-qPCR validation of histone modifications at the CDC6 promoter in 769P and Osrc-2 cells. Chromatin immunoprecipitation was performed using antibodies against H3, H3K4Me3, and H3K27Ac, with IgG as a negative control. Fold enrichment relative to input DNA is shown. (**C**,**D**) Cells were transfected with siRNAs targeting histone modifiers (SIRT3, SET1A, SET1B, HDAC6, KDM5B) or non-targeting siNC. CDC6 mRNA levels were measured by qRT-PCR. (**E**) Western blot analysis of CDC6 protein levels following knockdown of SET1A, SET1B, or SIRT3 in 769P, Osrc-2, 786-O. (**F**) SET1A knockdown reduces CDC6 and EXOSC5 protein expression in renal cancer cells in 769P, 786-O and Osrc-2 cells. Data are presented as mean ± SD (*n* = 3). *** *p* < 0.001.

To elucidate the mechanism underlying the upregulation of CDC6 in renal cell carcinoma tissues, we focused on epigenetic regulation and examined whether histone modifications play a role in regulating CDC6. Histone modification ChIP-seq data of ccRCC and normal renal tissues were downloaded from the GEO database to analyze the enrichment of transcription activation-related histone marks (H3K27ac and H3K4me3) at the CDC6 promoter. Compared with normal renal tissues, ccRCC tissues and primary ccRCC cells showed significantly higher H3K27ac and H3K4me3 enrichment at the CDC6 promoter (Figure 7A), indicating activated CDC6 transcription. ChIP-RT-PCR in 769P and Osrc-2 cells further confirmed H3K27ac and H3K4me3 enrichment at the CDC6 transcriptional initiation region (Figure 7B). To identify epigenetic enzymes regulating CDC6 histone modifications, integrated bioinformatics analysis was performed: (1) Epigenetic factors correlated with CDC6 expression were screened from the TCGA ccRCC dataset (epigenetic regulators list from http://www.epigeneticmachinery.org), with a Pearson correlation coefficient cutoff of >0.4 or <−0.4. UHRF1 showed the highest positive correlation with CDC6, while HDAC11 (a histone deacetylase) was negatively correlated (Supplementary Figure S3C). UHRF1 knockdown significantly downregulated CDC6 expression (Supplementary Figure S3D,E), which is the same as shown in prostate cancer [24], whereas HDAC11 knockdown had no obvious effect on CDC6 levels (Supplementary Figure S3F,G). Further analysis of methyltransferase/deacetylase expression and CDC6 correlation in the TCGA ccRCC dataset (Supplementary Figure S3A,B) showed no prominent correlation for most histone-modifying enzymes. We knocked down candidate enzymes specifically responsible for H3K4me3 and H3K27ac modifications in 769P cells. Our working hypothesis posited that the deacetylase SIRT3 and methyltransferase KDM5B negatively regulate CDC6, whereas the methyltransferases SETD1A and SETD1B exert positive regulatory effects on CDC6. Consistent with this hypothesis, SIRT3, SETD1A and SETD1B exhibited the expected regulatory patterns at the transcript level (Figure 7C,D). However, Western blot analysis (Figure 7E) showed that SIRT3 knockdown failed to upregulate CDC6 protein expression. In contrast, SETD1A and SETD1B positively modulated CDC6 at both the mRNA and protein levels. In this study, we focused on validating the regulatory relationship between SETD1A and CDC6. Knockdown of SETD1A in 769P, 786-O and Osrc-2 cells markedly reduced CDC6 expression (Figure 7F), indicating that SETD1A acts as a key epigenetic enzyme governing CDC6 modification and transcriptional regulation.

### 2.6. SETD1A Upregulates CDC6 Expression

**Figure 8:**
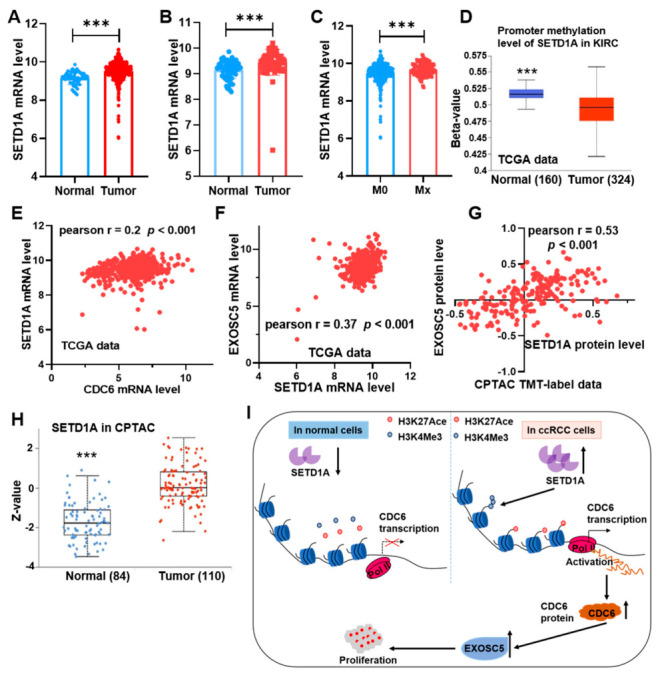
Clinical correlation of SETD1A expression and the proposed regulatory mechanism in ccRCC. (**A**) SETD1A mRNA levels were significantly upregulated in tumor tissues compared with normal tissues in TCGA. (**B**) Paired analysis of SETD1A expression in matched normal and tumor samples. (**C**) Elevated SETD1A expression was also observed in patients with distance metastasis patients. (**D**) Promoter methylation status of SETD1A in KIRC from the TCGA database. (**E**,**F**) Correlation analysis between SETD1A, CDC6, and EXOSC5 mRNA expression in the TCGA dataset. (**G**) A significant positive correlation was observed between SETD1A and EXOSC5 protein levels from CPTAC data (r = 0.53, *p* < 0.001). (**H**) SETD1A protein expression was significantly upregulated in tumor tissues (*n* = 110) compared with normal tissues (*n* = 84). (**I**) Schematic model of the proposed mechanism. In normal renal cells, SETD1A expression is low, leading to low levels of H3K4Me3 and H3K27Ac histone modifications at the CDC6 promoter, which maintains basal CDC6 transcription. In ccRCC cells, upregulated SETD1A increases active histone marks (H3K4Me3) at the CDC6 promoter, thereby enhancing CDC6 transcription. Elevated CDC6 protein further activates the CDK2-EXOSC5 axis, ultimately promoting ccRCC cell proliferation, invasion, and migration. *** *p* < 0.001.

We further investigated the potential clinical relevance of SETD1A in clear cell renal cell carcinoma. SETD1A was significantly upregulated at both the mRNA (Figure 8A) and protein levels (Figure 8H) in tumor tissues compared with non-tumor tissues, and this upregulation trend was consistent in 72 paired tumor-normal tissue samples (Figure 8B). Additionally, SETD1A expression was significantly elevated in patients with distant metastasis (Figure 8C), suggesting its potential association with tumor progression. In tumor tissue samples, the methylation modification level at the promoter region of SETD1A was significantly increased (Figure 8D). We then analyzed the expression correlation between SETD1A and CDC6, and the results demonstrated a positive correlation at the mRNA level (Figure 8E). Given the positive regulation of EXOSC5 by CDC6, we further explored the correlation between SETD1A and EXOSC5. The results showed that SETD1A was positively correlated with EXOSC5 at both the mRNA and protein levels (Figure 8F,G). Western blotting results further confirmed that knockdown of SETD1A markedly downregulated EXOSC5 expression in 769P, 786-O and Osrc-2 cells (Figure 7F). Collectively, these findings demonstrate that SETD1A is highly expressed in ccRCC and positively regulates CDC6 expression (Figure 8I).

## 3. Discussion

Clear cell renal cell carcinoma (ccRCC), the predominant subtype of renal cell carcinoma (RCC), remains one of the most aggressive and therapy-resistant malignancies of the urinary system [1,2,3]. Despite advances in targeted therapy, the molecular mechanisms driving ccRCC progression are still not fully elucidated. Cell division cycle 6 (CDC6), a key licensing factor for DNA replication, forms the pre-replication complex (pre-RC) by cooperating with ORC, CDT1, and the MCM2-7 helicase (our IP-MS data identified MCM5 and MCM7 as interaction partners of CDC6, as shown in Supplemental Table S2) to ensure accurate cell-cycle progression [8,9]. Beyond its canonical role in pre-RC assembly and G1/S transition, CDC6 possesses well-established anti-apoptotic functions. Mechanistically, it binds activated Apaf-1 and sterically hinders apoptosome formation, thereby elevating the threshold for intrinsic apoptosis activation [36]. Attenuation of CDC6 in ccRCC cells leads to a marked suppression of proliferation and migration, induction of apoptosis, and impaired tumor growth in vivo [27]. CDK2 is a known kinase reported to phosphorylate CDC6 [35]. In our data, CDC6 depletion led to decreased CDK2 expression in 769P and OSRc-2 cells (Supplementary Figure S1B,C). We attribute the observed reduction in CDK2 to a secondary effect of cell cycle arrest rather than direct regulation, as CDC6 lacks inherent transcriptional activity. Mechanistically, CDC6 depletion likely triggers replication stress or activates the intra-S phase checkpoint, leading to impaired CDK2 activation and G1 arrest. Thus, the lower CDK2 levels primarily reflect a contraction of the proliferative pool [37]. Notably, we acknowledge the complexity of this regulation. CDC6 has been reported to support CDK2 activity through an ATP-dependent physical interaction that displaces p21/p27 from Cdk2–cyclin complexes [36], and to modulate ATR-Chk1 checkpoint tone [38]. This raises the possibility of a partially direct contribution to CDK2 activity state. However, deciphering the precise regulatory relationship between CDK2 and CDC6 will require further experimental validation.

Our clinical analyses validated that CDC6 is significantly overexpressed in more than 97% of ccRCC tissues compared with adjacent normal tissues (Figure 1C,D) and accurately predicts lymph node metastasis (Figure 1I) and poor overall survival (Figure 1K–M), supporting its utility as a diagnostic and prognostic biomarker. Functionally, knockdown of CDC6 markedly suppressed proliferation in multiple ccRCC cell lines, including 769P and OSRC-2. This proliferative phenotype was recapitulated in 786-O and ACHN cells (Supplemental Figure S2), which is consistent with a previous study, and in vivo tumor formation assays have demonstrated that CDC6 knockdown significantly suppresses tumor growth [27]. These results firmly establish CDC6 as a bona fide oncoprotein that drives ccRCC growth. To explore downstream mechanisms, we performed unbiased label-free quantitative proteomics and immunoprecipitation-mass spectrometry (IP-MS), identifying EXOSC5/G3BP2/RPL15 as potential downstream targets and interaction partners of CDC6 (Figure 4H,I and Figure 5C,D). Following validation by RT-PCR and Western blotting, we identified that EXOSC5 and G3BP2 were regulated by CDC6 (Figure 5A–D). EXOSC5 is an essential subunit of the RNA exosome complex, which mediates RNA processing and degradation and has been implicated in cancer stemness, proliferation, and chemoresistance in endometrial, colorectal, and gastric cancers [28,29,30]. The expression and function of EXOSC5 in ccRCC have not been reported. Our data demonstrated that EXOSC5 is significantly upregulated in ccRCC at both mRNA and protein levels (Figure 6B,H). Knockdown of CDC6 reduced EXOSC5 expression in multiple ccRCC cell lines (Figure 5E,F), CDC6 interacts with EXOSC5, as verified by affinity MS (Figure 4H and Supplemental Table S2). In addition, our data indicate that CDC6 knockdown is accompanied by a slight upregulation of G3BP2 across multiple ccRCC cell lines. The G3BP family, comprising G3BP1 and G3BP2, contains RNA-binding proteins crucial for RNA stabilization, protein localization, and stress granule formation [39,40]. These findings suggest that, beyond its canonical role in cell cycle regulation, CDC6 may also participate in RNA processing and degradation, potentially through modulating RNA-binding proteins crucial for RNA stabilization and protein localization. However, the direct interaction between CDC6 and EXOSC5 (or G3BP2), coupled with the impact of EXOSC5 on malignant phenotypes, requires further validation. Specifically, comprehensive rescue assays are needed to definitively determine whether EXOSC5 is a direct functional target of CDC6.

Epigenetic dysregulation, especially histone modifications, plays a critical role in ccRCC development. Public ChIP-seq data and experimental ChIP-qPCR revealed significant enrichment of H3K4me3 and H3K27ac—two histone marks associated with active transcription—at the CDC6 promoter in ccRCC tissues and cells compared with normal controls. Screening of epigenetic modifiers identified SETD1A, a histone H3K4 methyltransferase belonging to the KMT2 family, as a potential upstream regulator responsible for CDC6 transactivation. SETD1A is frequently dysregulated in human cancers and promotes tumor progression by methylating H3K4 and activating oncogene transcription. In this study, SETD1A was significantly upregulated in ccRCC (Figure 8A,H) and positively correlated with CDC6 (Figure 8E) and EXOSC5 (Figure 8F,G) expression. Knockdown of SETD1A markedly suppressed CDC6 and EXOSC5 expression (Figure 7F). Notably, this regulatory axis has not been previously reported in ccRCC, highlighting the novelty of our findings. However, direct evidence demonstrating that SETD1A regulates the methylation of specific regions within the CDC6 promoter, as well as the identity of the enzymes governing CDC6 promoter acetylation, remains to be further validated by ChIP assays.

In summary, our study demonstrates that CDC6 is activated by SETD1A and functions as an oncogenic driver in ccRCC by regulating the downstream protein EXOSC5. The newly identified SETD1A–CDC6–EXOSC5 signaling axis plays a critical role in promoting ccRCC proliferation and progression and serves as a novel diagnostic and prognostic biomarker. These findings deepen our understanding of the epigenetic and post-transcriptional mechanisms underlying ccRCC pathogenesis and provide a theoretical foundation for developing novel diagnostic and prognostic interventions for this lethal malignancy.

## 4. Material and Method

### 4.1. Cell Culture

All cells were generously provided by the research group of Professor Zesong Li from the Guangdong Provincial Key Laboratory of Systems Biology and Synthetic Biology for Urogenital Tumors. Cells were cultured in a humidified incubator at 37 °C with 5% CO_2_. 786-O, 769P, and Osrc-2 cells were maintained in RPMI 1640 medium; 293T cells in Dulbecco’s modified Eagle’s medium (DMEM); ACHN and HK-2 cells in Eagle’s MEM or DMEM/F12 medium; Caki-2 cells in McCoy’s 5A medium. All culture media were obtained from Gibco. All media were supplemented with 10% fetal bovine serum (FBS; Gibco, Grand Island, NY, USA) and 1% penicillin–streptomycin (Gibco).

### 4.2. Plasmids, shRNAs, siRNAs Transfection

The full-length human CDC6 cDNA was cloned into the pCMV-Flag vector to generate the Flag-tagged CDC6 overexpression plasmid. Empty pCMV-Flag vector served as the negative control (oe-NC). Small interfering RNAs (siRNAs) targeting CDC6, SETD1A, SIRT3, SETD1B, HDAC6, KDM5B, UHRF1, HDAC11, and non-targeting siRNA (siNC) were designed and synthesized by GenePharma (Shanghai, China). All siRNAs were used at a final concentration of 60 nM. Short hairpin RNAs (shRNAs) targeting CDC6 and a non-targeting shNC were cloned into the GV493 lentiviral vector. All sequences are listed in Table 1. Transient transfections of plasmids and siRNAs were performed using Lipofectamine 3000 reagent (Thermo Fisher Scientific, Waltham, MA, USA, L3000015) according to the manufacturer’s instructions. Briefly, cells were transfected in Opti-MEM medium for 6 h, then cultured in complete medium for an additional 48 h before collection for subsequent experiments.

### 4.3. CCK8 Cell Proliferation Assay

Cells were seeded into 96-well plates (2000–3000 cells/well) after transfection. Cell viability was measured daily using the Cell Counting Kit-8 (CCK-8; NCM Biotech, Suzhou, China, C6005). Absorbance at 450 nm was detected using a microplate reader.

### 4.4. Colony Formation Assay

Cells were plated in 6-well plates (1000–1500 cells/well) and cultured for 14 days. Each well was washed with PBS twice, then fixed with methanol for 15 min, stained with 0.5% crystal violet solution for 20 min and manually counted.

### 4.5. 5-Ethynyl-2′-deoxyuridine Incorporation Assay (EDU)

Cell proliferation was assessed using the EdU Assay Kit (Ribobio, Guangzhou, China, Cell-Light EDU Apollo567 In vitro Kit, C10310-1). Transfected cells were incubated with 50 μM EdU for 2 h, fixed, permeabilized, and stained with Apollo^®^ fluorescent dye. Nuclei were counterstained with Hoechst. Images were captured using a fluorescence microscope, and the percentage of EdU-positive cells was calculated using the formula: (Number of EdU-positive cells/Total number of Hoechst-stained nuclei) × 100%. For each experimental condition, at least 3 random microscopic fields were captured per well, and a total of 100–200 cells were counted in total.

### 4.6. RNA Isolation and Quantitative Real-Time PCR (qRT-PCR)

Total RNA was extracted from cultured cells using TRIzol reagent (Thermo Fisher Scientific, Waltham, USA, 15596026). RNA concentration and purity were assessed using a NanoDrop spectrophotometer (Thermo Fisher Scientific). The isolated total RNA was reverse transcribed into cDNA using the mRNA RT Reagent Kit (Accurate Biology, Changsha, China, AG11728). The synthesized cDNA was used in the RT-qPCR assay using the SYBR Green Pro Taq HS (Accurate Biology, AG11701) on a Roche LightCycler 480 Fast Real-Time PCR Detection System. Relative gene expression was calculated using the 2^−ΔΔCt^ method, with ACTIN as the internal reference. Primer sequences are provided in Table 2.

### 4.7. Western Blotting

Cells were lysed in ice-cold RIPA lysis buffer supplemented with protease and phosphatase inhibitor cocktails. Protein concentrations were determined using the BCA Protein Assay Kit (Thermo Fisher Scientific). Equal amounts of protein (10–20 μg) were separated by 5–12% SDS-PAGE and transferred to PVDF membranes (Immobilon^®^ -P PVDF Membrane, Merck Millipore, Hessen, Germany, IPVH00010). Membranes were blocked with 5% non-fat milk in TBST for 1 h at room temperature, then incubated with primary antibodies overnight at 4 °C. After washing, membranes were incubated with HRP-conjugated secondary antibodies for 1 h at room temperature. Protein bands were visualized using Tanon-5200Muti (version, 1.0.0.0). The resulting images were quantified using the ImageJ software (version, 1.51j8). The following antibodies were used: CDC6 (Proteintech, Wuhan, China, 66021-1-AP, 11640-1-AP), CDK2 (Proteintech, 10122-1-AP), EXOSC5 (Proteintech, 15627-1-AP), Tubulin (Proteintech, 66031-1-AP), GAPDH (Proteintech, 60004-1-AP), β-actin (Proteintech, 81115-1-AP), and RPL15 (Proteintech, 16740-1-AP), G3BP2 (Proteintech, 16276-1-AP), SETD1A (Proteintech, 67936-1-lg). Regarding the choice of loading controls: The initial CDC6 antibody we used (Proteintech, 66021) yielded a signal around 70 kDa. Based on our protein marker, which clearly distinguishes the 50 and 70 kDa bands, α-Tubulin (~55 kDa) served as an appropriate loading control. In subsequent experiments, we compared both Tubulin and GAPDH as internal references and confirmed that neither control affected the quantitative outcome. Both controls were retained in the relevant figures to document the basis for our reference selection. Later, we switched to another CDC6 antibody (Proteintech, 11640) that detects a band below 70 kDa. Consequently, we adopted GAPDH as the loading control to better resolve the CDC6 and GAPDH bands. In Figure 7E, we simultaneously probed for CDK2, whose molecular weight is close to that of GAPDH; therefore, β-Actin was used instead of GAPDH as the loading control.

### 4.8. Clinical Tissue Samples

Four pairs of ccRCC tissues and matched adjacent normal tissues were collected from patients admitted to the First Affiliated Hospital of Shantou University Medical College. All participants provided written informed consent. The study was conducted in accordance with the Declaration of Helsinki, and the protocol was approved by the Ethics Committee of the First Affiliated Hospital of Shantou University Medical College (number: 2020-031). All tissue samples were obtained directly from surgery after tissue removal for routine pathological examination and confirmed for ccRCC. All tissue samples were immediately flash-frozen in liquid nitrogen and subsequently stored at −80 °C.

### 4.9. Label-Free Quantitative Proteomics

OSRC-2 cells transfected with siNC or siCDC6 were collected for label-free quantitative proteomics. Cells were washed three times with cold PBS and then lysed in SDT lysis buffer (0.2% SDS (*m*/*v*), 100 mM DTT, 100 mM Tris, pH = 7.6). After centrifugation, the supernatant was collected for protein concentration determination. A total of 100 μg protein per sample was taken for in-solution digestion as described previously [41]. Subsequently, detection was performed using an Orbitrap Exploris 480 mass spectrometer Data-Dependent Acquisition (DDA) model. Data were processed using PD software (version, 2.4) with FDR < 0.01. Differentially expressed proteins (DEPs) were defined as fold change (FC) > 2 or <0.5 with *p* < 0.05.

### 4.10. Co-Immunoprecipitation (Co-IP) and Mass Spectrometry (IP-MS)

Immunoprecipitation was performed as described previously with slight modifications [42]. The Flag-beads were purchased from Thermo Fisher Scientific (A36797). For Co-IP assays, 293T cells were transfected with Flag-CDC6 or an empty vector. After 48 h, cells were lysed in IP lysis buffer containing protease inhibitors. Whole-cell lysates were incubated with anti-Flag beads at 4 °C for 4 h with gentle rotation. Beads were washed four times with lysis buffer, then boiled in 2× SDS loading buffer for 5 min. Immunoprecipitated proteins were analyzed by Western blot or subjected to mass spectrometry (MS). For IP-MS, immunoprecipitated proteins were separated by SDS-PAGE, visualized by Coomassie blue staining, and in-gel digested with trypsin, subsequently detected by 480 mass spectrometers.

### 4.11. Chromatin Immunoprecipitation (ChIP) Assay

ChIP assays were performed using the SimpleChIP^®^ Plus Enzymatic Chromatin IP Kit (Magnetic Beads) (Cell Signaling Technology, Danvers, MA, USA, #9005) according to the manufacturer’s instructions. Briefly, cells were cross-linked with 1% formaldehyde for 10 min at 37 °C, quenched with glycine, and lysed. Chromatin was sheared into 200–500 bp fragments by sonication. Soluble chromatin was immunoprecipitated with antibodies against H3K4me3 (Proteintech, 39060), H3K27ac (Proteintech, 39034), total H3 (including in the Kit, 4620), or control IgG (including in the Kit, 2729) at 4 °C overnight. Immunocomplexes were captured with Protein G Magnetic Beads (including in the Kit, 9006), washed, and eluted. Cross-links were reversed at 65 °C overnight, and DNA was purified. Enrichment at the CDC6 promoter region was analyzed by qRT-PCR. Primers of CDC6 were listed in Table 3.

### 4.12. Bioinformatics Analysis

The gene expression profiles and clinical data of patients with ccRCC were obtained from UCSC Xena (https://xenabrowser.net/datapages/, accessed on 27 December 2020), including 533 ccRCC cases in TCGA and the protein expression profiles were obtained from CPTAC (https://proteomics.cancer.gov/programs/cptac, accessed on 1 November 2020). The UALCAN online tools were used to analyze the protein level pattern (https://ualcan.path.uab.edu/analysis.html, accessed on 3 March 2026). Protein–protein interaction analysis was conducted on the STRING website (https://string-db.org/, accessed on 18 March 2023), and the results were visualized and refined using Cytoscape software (version 3.10.4). Pearson correlation analysis was performed to evaluate correlations between gene expression levels. Kaplan–Meier survival curves for overall survival (OS), disease-specific survival (DSS), and progression-free interval (PFI) were plotted using the log-rank test by GraphPad Prism 8.0 (GraphPad Software, Inc., San Diego, CA, USA). Time-dependent receiver operating characteristic (ROC) curves were generated to evaluate the predictive accuracy of the prognostic model using SPSS 24.0. (IBM SPSS, Chicago, IL, USA). ChIP-seq data of KIRC samples and normal tissues were obtained from the NCBI Gene Expression Omnibus (GEO) datasets (https://www.ncbi.nlm.nih.gov/gds/, accessed on 30 July 2022). The BED files were visualized using the Integrative Genomics Viewer (IGV). The information on ChIP-seq data was listed in Table 4.

## Figures and Tables

**Table 1:** SiRNA and shRNA sequences.

Gene Name	Sense (5′-3′)
siCDC6-1	AGGCACUUGCUACCAGCAATT
siCDC6-2	CCAAGAAGGAGCACAAGAUTT
siCDC6-3	GACAAUCAGCUGACAAUUATT
ShCDC6-1	GCTGTTGAACTTCCCACCTTA
ShCDC6-2	CCAGCTATTGCTCAGGAGATT
ShCDC6-3	CGGGCATTGAACAAAGCTAAA
siSIRT3	GCUUCAAGUGUUGUUGGAA
SiSET1A	GCUUGACAUCAAAGGACAA
SiSET1B	GCAUCGAGAUGCUGCUGAA
SiHDAC6	GGUGUUGGAUGAGCAGUUA
SiKDM5B	GCAGUUGUUUGCAAGGAUA

**Table 2:** RT-PCR primers.

Gene Name	F Primer	R Primer
CDC6-1	AAGGGCGTTGGGGTCATAAG	GGCTTCATCTAAGGGCAGCA
CDC6-2	AACCAAACCCAGAAGGCTCT	AACCAAACCCAGAAGGCTCT
CDC6-3	TGCCCTTAGATGAAGCCACT	TATAAGGGTGCCCTCTGGTGT
SIRT3	CCCCAAGCCCTTTTTCACTTT	CGACACTCTCTCAAGCCCA
SET1A	CAGAAGGTGTACCGCTATGATG	TCTTGGAGGTCTTCGACTGGT
SET1B	AGGGGCATCATAAACTGTACCG	GGGGATCTTCGACAATTTCCAC
HDAC6	GCGGGGAAAAGGTCGC	CTGGCCGGTTGAGGTCAT
KDM5B	AGTGGGCTCACATATCAGAGG	CAAACACCTTAGGCTGTCTCC
G3BP2	GAGCTGAAACCACAAGTGGAGG	GGTCACTGAAGCCCAGGAGAAA
RPL15	ACAAGGCCAAGCAAGGTTAC	ACTGAAGGCTTCGAGCAAAC
EXOSC5	CCGGCACTTTGCCTGCGAAC	GAAGAGAGCCCGCATGGGCA
ACTIN	CATCCGCAAAGACCTGTACG	CCTGCTTGCTGATCCACATC

**Table 3:** CDC6 primer for Chip-seq qPCR.

Gene Name	F Primer	R Primer
CDC6-promoter +0.2 kb	CAGTTATGCGTGGTGTGAAGG	AGCACCCGCCACATTTAGTC
CDC6-promoter −0.2 kb	CCCGCTTTACCCAGAGTCG	ACAGAGCCTTTCGCCTTGG

**Table 4:** GEO data information.

DataSet	Antibody	Sample Type	Tissue No.
GSM2293421	H3K27Ace	Normal tissue	12364284
GSM2293314	H3K27 Ace	Primary cells	86049102
GSM2293430	H3K27 Ace	Tumor tissue	40911432
GSM2293319	H3K27 Ace	Primary cells	40911432
GSM2293423	H3K4Me3	Normal tissue	12364284
GSM2293315	H3K4Me3	Primary cells	40911432
GSM2293436	H3K4Me3	Tumor tissue	86049102
GSM2293310	H3K4Me3	Primary cells	86049102

## Data Availability

The original contributions presented in this study are included in the article/Supplementary Material. Further inquiries can be directed to the corresponding authors.

## Supplementary Materials

The following supporting information can be downloaded at: https://www.mdpi.com/article/10.3390/ijms27135657/s1.
