# Supplementary material for: H3K4me3 and H3K27ac Promote ccRCC Proliferation Through the CDC6-EXOSC5 Axis
Source: Int J Mol Sci. 2026 Jun 23;27(13):5657. doi: 10.3390/ijms27135657 (PMC13362089; doi:10.3390/ijms27135657)
Supplement: Supplementary file 1 [file ijms-27-05657-s001.zip › Supplementary Figures.pdf]

Supplementary figure and legend

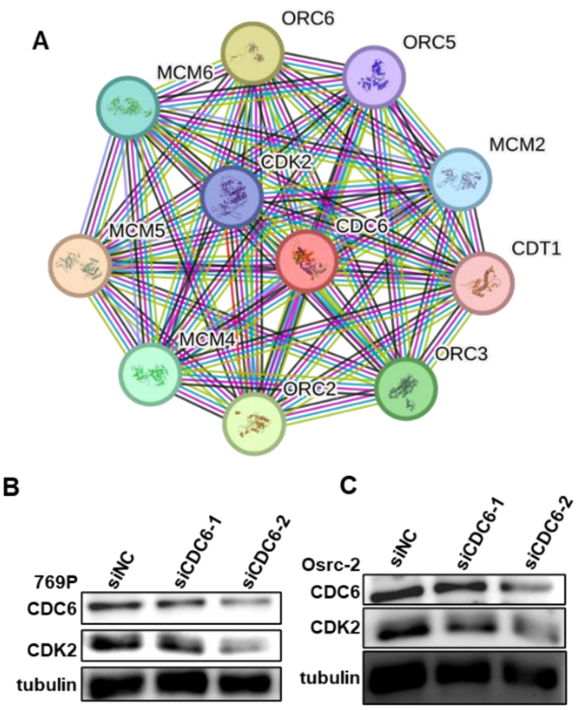

Supplementary Figure S1. CDC6 interacts with replication-related proteins and regulates CDK2 expression. (A) Protein-protein interaction (PPI) network analysis of CDC6 and its associated replication factors. The network illustrates the interactions between CDC6 and key components of the pre-replication complex, including CDK2, ORC family proteins (ORC2/3/5/6), MCM family proteins (MCM2/4/5/6), and CDT1. (B, C) Western blot analysis of CDK2 expression following CDC6 knockdown in 769P (B) and Osrc-2 (C) cells.

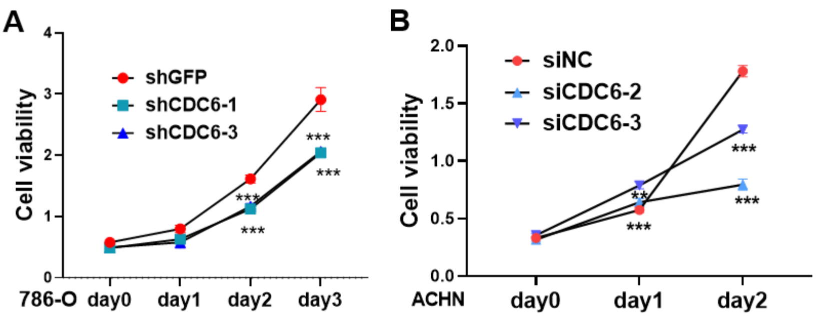

Supplementary Figure S2. CDC6 knockdown inhibits renal cancer cell viability. Cell viability was measured by CCK-8 assay in 786-O (A) and ACHN (B) cells after CDC6 knockdown. Data are presented as mean ± SD (*n*=5). ***$p < 0.001$.

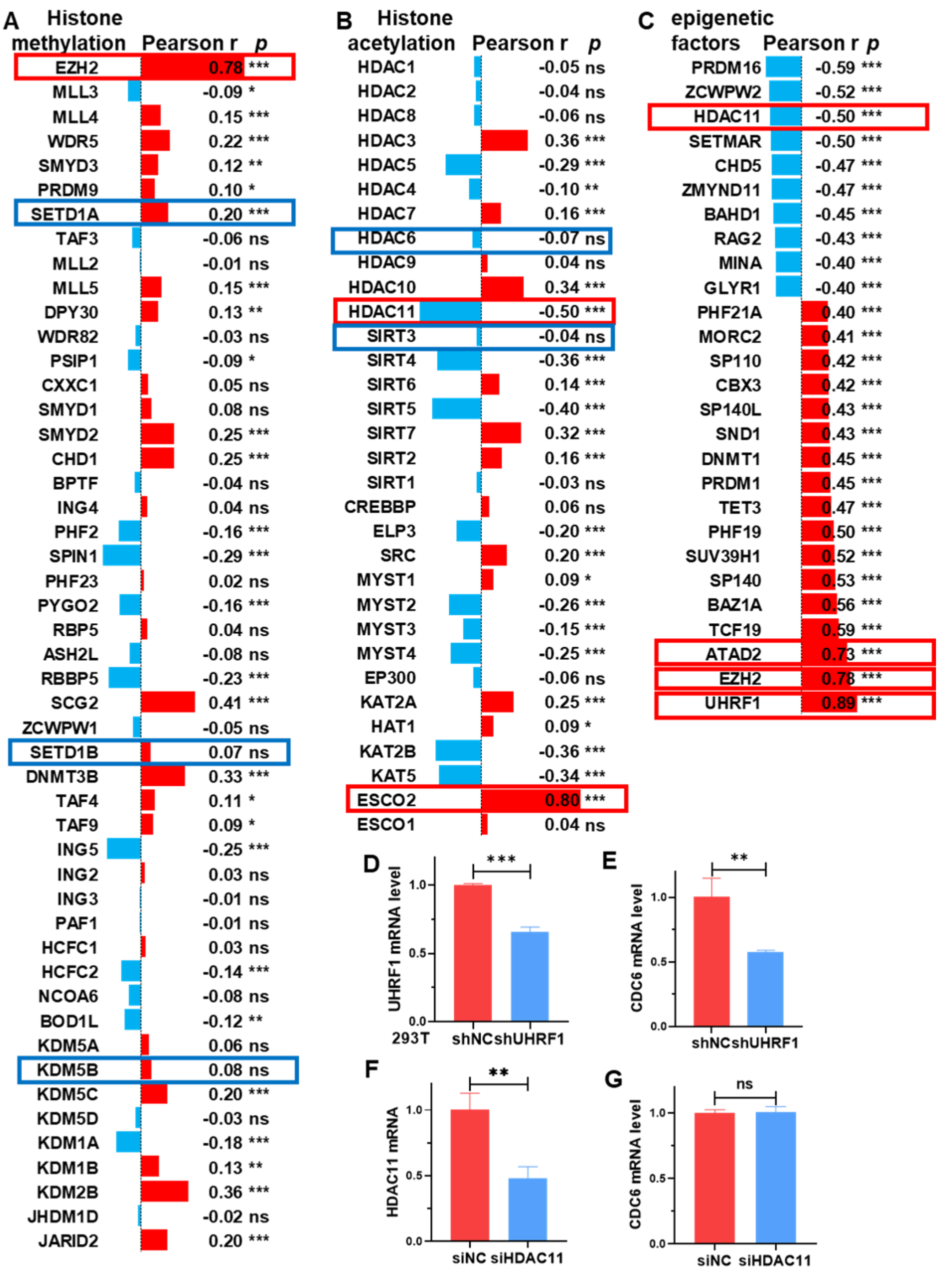

Supplementary Figure S3. Correlation analysis of CDC6 with epigenetic regulators and validation of key hits. (A–C) Pearson correlation analysis between CDC6 mRNA expression and the expression of genes involved in histone methylation (A), histone acetylation (B), and other epigenetic regulators (C) in the TCGA dataset. (D) qRT-PCR validation of UHRF1 knockdown efficiency in 293T cells transfected with shNC or shUHRF1. (E) CDC6 mRNA levels following UHRF1 knockdown in 293T cells. (F) qRT-PCR validation of HDAC11 knockdown efficiency in cells transfected with siNC or siHDAC11. (G) CDC6 mRNA levels following HDAC11 knockdown, showing no significant change. Data are presented as mean ± SD (n=3). *$p < 0.05$; **$p < 0.01$; ***$p < 0.001$. ns, not significant.
